# In-hospital severe COVID-19 in a philanthropic tertiary hospital setting: is asthma a concern? A retrospective study

**DOI:** 10.1590/1516-3180.2021.0403.R2.15122021

**Published:** 2022-08-01

**Authors:** Gabriela Accetta Rojas, Flávia Nascimento Ost, Roberto Stirbulov, Ozíris Simões

**Affiliations:** I Medical Student, Faculdade de Ciências Médicas da Santa Casa de São Paulo (FCMSCSP), São Paulo (SP), Brazil.; II Medical Student, Faculdade de Ciências Médicas da Santa Casa de São Paulo (FCMSCSP), São Paulo (SP), Brazil.; III MD, PhD. Full Professor of Internal Medicine, Faculdade de Ciências Médicas da Santa Casa de São Paulo (FCMSCSP), São Paulo (SP), Brazil; and Clinical Chief, Irmandade da Santa Casa de Misericórdia de São Paulo (ISCMSP), São Paulo (SP) Brazil.; IV MD, PhD. Assistant Professor, Collective Health Department, Faculdade de Ciências Médicas da Santa Casa de São Paulo (FCMSCSP), São Paulo (SP), Brazil.

**Keywords:** Asthma, COVID-19, SARS-CoV-2, Hospitalization, Death, Severe COVID-19, Philanthropic tertiary hospital, Need for intubation

## Abstract

**BACKGROUND:**

The frequency of coronavirus disease 2019 (COVID-19) cases among asthmatics has been reported to be reduced. However, the findings regarding the association between asthma and the risk of severe COVID-19 have been divergent.

**OBJECTIVE:**

To investigate whether asthma is associated with a reduced risk of development of severe COVID-19.

**DESIGN AND SETTING:**

Retrospective analysis on COVID-19 surveillance databases at two tertiary-level hospitals in São Paulo, Brazil.

**METHODS:**

The medical records of patients hospitalized due to COVID-19 between March and August 2020 were reviewed in accordance with the clinical, laboratorial, radiological and epidemiological criteria for COVID-19, and for comorbidities.

**RESULTS:**

Among the adult patients included (> 18 years of age) there were 52 asthmatics and 1,318 non-asthmatics. Their median ages and interquartile ranges (IQR) were, respectively, 54 (41-69) and 60 (44-72) years. At least one comorbidity was seen in 73% of asthmatics and 56% of the non-asthmatics. Among the asthmatics, most presented mild asthma (92%) and the prevalence of chronic obstructive pulmonary disease (COPD) was high (27%). The asthmatics presented an unadjusted odds ratio (OR) for severe COVID-19 of 0.89 (95% confidence interval, CI 0.5-1.56); and OR 0.88 (95% CI 0.5 -1.68) after multivariable adjustment. Age > 60 years, male sex, hypertension, diabetes, cancer and homelessness were covariates associated with increased odds for severe COVID-19. Kaplan-Meier estimated survival over hospitalization of up to 30 days did not differ between the groups (log-rank P = 0.09).

**CONCLUSIONS:**

The association between asthma and decreased risk of severe COVID-19 or increased survival was statistically non-significant.

## INTRODUCTION

Asthma is characterized as a disease involving chronic airway inflammation and variable limitation of airflow. In asthmatics, upper-airway viral infection is a well-known risk factor for symptom exacerbations and hospitalizations. Severe acute respiratory syndrome coronavirus 2 (SARS-CoV-2), a novel virus that mainly affect the airways, has not clearly followed this pattern. Among hospitalized patients with coronavirus disease 2019 (COVID-19), the prevalence of asthma has been low and in some cohorts it has been reported that asthma was a condition that gave rise to reduced risk of severe COVID-19 presentations,^1,2^ differently from other chronic lung diseases.^3^

Reduced expression of angiotensin-converting enzyme 2 (ACE2), identified as one of the key receptors for viral cellular entry,^4^ has been observed in asthmatics. Use of inhaled corticosteroids and the allergic asthma phenotype seem to be the main underlying mechanisms for reduced ACE2 expression,^5,6^ thus providing a plausible causal explanation for decreased risk of severe COVID-19 among asthmatics. However, to date, the findings from observational studies regarding asthma and severe COVID-19 remain divergent. Additionally, to our knowledge, no studies have addressed this relationship in the context of a highly socially vulnerable population.

## OBJECTIVE

Therefore, the aim of this study was to investigate the association between asthma and development of severe COVID-19 and in-hospital mortality in a unique tertiary-level hospital complex that cares for the underserved population in the largest Latin American city.

## METHODS

### Study design

We conducted a retrospective study in a medical center formed by two tertiary-level hospitals, through access to the hospitals’ COVID-19 surveillance databases. The electronic medical records of all hospitalized patients with acute respiratory symptoms who were suspected of having COVID-19 and who were admitted between March 1 and August 28, 2020, were reviewed. These two hospitals share the same location and staff, and differ mainly with regard to healthcare access: one is restricted to health-insured individuals, while the other is a philanthropic hospital, providing care mainly to uninsured patients. The COVID-19 surveillance database is composed of cases that were evaluated in the emergency department and were found to present suspected symptoms of COVID-19. Some of these patients required hospitalization, while others developed COVID-19 while hospitalized. This study was conducted in accordance with the ethical principles embodied in the Declaration of Helsinki and local applicable laws and regulations. It was approved by the Human Research Ethics Committee of the Irmandade da Santa Casa de Misericórdia de São Paulo (CAEE no. 36961020.9.0000.5479) on September 11, 2020.

### Study population

The institutional criteria for hospital admission were two or more of the following: respiratory rate ≥ 22 breaths/minute or dyspnea, oxygen saturation < 95% in room air, fever and alterations seen on computed tomography (CT) of the chest.

The electronic medical records of individuals identified in the COVID-19 surveillance database were reviewed regarding their hospitalization. Patients ≥ 18 years of age were included if they presented at least three of the following findings: clinical criteria in accordance with the case definition of the Centers for Disease Control and Prevention (CDC),^7^ epidemiological criteria, radiological criteria^8^ and positive real-time polymerase chain reaction (RT-PCR) for SARS-CoV-2. Patients with unknown clinical outcomes were excluded.

### Covariates

Age was categorized into the following groups: 18 to < 40, 40 to < 50, 50 to < 60, 60 to < 70, 70 to < 80 and ≥ 80 years. Regarding comorbidities, we extracted from the records those that have been associated with higher risk of severe illness and mortality, such as hypertension, diabetes, chronic cardiac disease (heart failure, previous myocardial infarction or arrhythmias), chronic kidney disease, obesity, current smoking, active cancer, chronic obstructive pulmonary disease (COPD), chronic lung disease (other than COPD and asthma) and immunosuppression (human immunodeficiency virus (HIV)-positive individuals and transplant recipients). In addition, we created two other binary variables: regular use of inhaled corticosteroids (ICS) and homelessness. ICS is part of the treatment therapy for asthma and COPD, and has been indicated as a possible protective factor against COVID-19 severity. Thus, in this study, it was included as an important confounder. In the context of a philanthropic hospital, we included homelessness as a proxy variable for extreme social vulnerability.

Furthermore, epidemiological week was used as a covariate to account for possible unmeasured effects regarding changes to transmission dynamics, resource availability and standard of care development, given the course of the pandemic. The first epidemiological week in the analysis was determined as the week of the first in-hospital case registered.

Asthma was categorized into two groups: either mild or moderate to severe, according to the treatment step and following current Global Initiative for Asthma (GINA) classification.^9^ Patients whose medical records did not report any continuous use of medications for asthma and only use of asthma reliever therapy, and those whose daily use consisted only of low-dose inhaled corticosteroids, were considered to be mild asthmatics. Patients with at least medium to high-dose use of inhaled corticosteroids were considered to have moderate to severe asthma. Possible optimization of asthmatic therapy during hospitalization was not taken into account for this classification, which only considered previous pharmacological therapy. as reported in the medical records at the time of admission.

### Outcomes

We considered that severe COVID-19 was a binary composite outcome, comprising either death or the need for intubation. In the Cox proportional-hazards model, death was considered to be the outcome.

### Statistical analysis

Descriptive baseline characteristics were reported as percentages when they were categorical variables, or as medians and interquartile ranges when they were continuous variables. No sample size calculation was performed, given that the size of the database was considered fixed.

A logistic regression model was used to estimate the association between severe COVID-19 and asthma. The model was adjusted for age > 60, sex, regular ICS use, chronic cardiac disease, diabetes, hypertension, COPD, chronic lung disease, active cancer, obesity, immunosuppression, smoking and homelessness.

Kaplan-Meier curves were constructed to show the cumulative probability of survival over hospitalization of up to 30 days. The survival functions were compared using a log-rank test.

**Figure 1.**
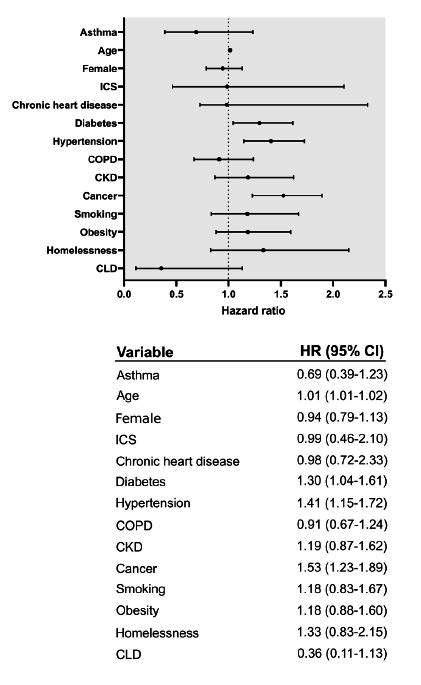
Risk of death due to COVID-19.

**Figure 2.**
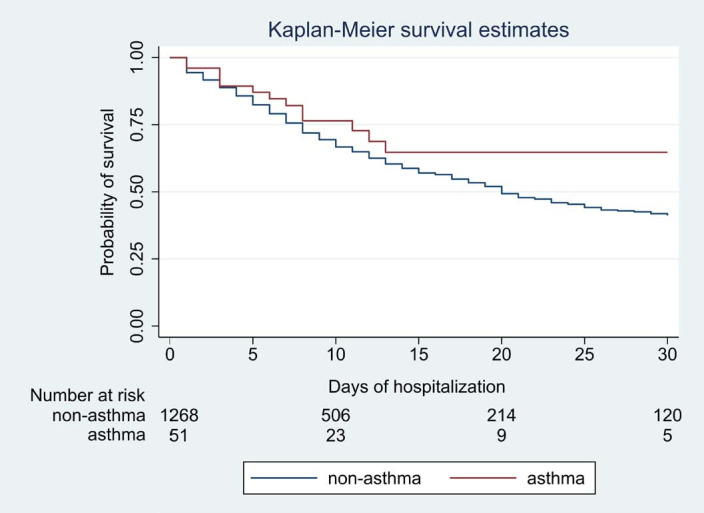
Survival over 30 days of hospitalization.

We calculated hazard ratios (HR) for death according to the duration of hospitalization for asthmatics and non-asthmatics, through use of a Cox proportional-hazards model adjusted for age, sex, regular ICS use, chronic heart disease, diabetes, hypertension, COPD, chronic lung disease, chronic kidney disease, cancer, obesity, smoking and homelessness. Testing of the proportional-hazards assumptions was performed. Statistical significance was accepted with a 95% confidence interval (CI) and P values < 0.05. GraphPad Prism version 8 (GraphPad, La Jolla, California, United States) was used to elaborate Figure 1. Stata version 16 (StataCorp, Texas, United States) was used for the statistical analyses and to elaborate Figure 2.

HR = hazard ratio; CI = confidence interval; COPD = chronic obstructive pulmonary disease; ICS = inhaled corticosteroids.

## RESULTS

We assessed the data of all 1,529 hospitalized individuals, except for 76 individuals under 18 years of age and 83 for whom data regarding either the COVID-19 diagnosis or related death were missing. Among the 58 individuals registered as asthmatics, 52 were included as asthmatics and six were considered to be non-asthmatics because of conflicting data.

The asthmatics represented 3.8% of the hospitalized patients with COVID-19. A slightly higher proportion of women (60%) was seen in the asthma group, in comparison with non-asthmatics (41.3%). The median age and interquartile range (IQR) were 54 (41-69) years among asthmatics and 60 (44-62) years among non-asthmatics. Most of the individuals in both groups were ≥ 60 years old, and the age distribution over the decades did not present any important variation between the groups. Patients < 50 years old were proportionally more prevalent in the asthma group.

The prevalence of comorbidities was remarkably high in both groups, such that 73% of the asthmatics and 57% of the non-asthmatics presented at least one comorbidity. Hypertension was the most common comorbidity in both groups (38% in the asthma group versus 31% in the non-asthma group), followed by diabetes. A minimal difference was seen regarding diabetes: 21% versus 18.6%, respectively. COPD was particularly high in the asthma group: 14 (27%) versus 103 (8%) in the non-asthma group. Use of ICS, either as reliever therapy or daily therapy, was seen in 85% of asthmatics. Mild asthma was present in 48 asthmatics (92%) (Table 1).

**Table 1.** Demographic variables

Variable	Asthmatics (%) n = 52	Non-asthmatics (%) n = 1,318
**Age (median, IQR)**	54 (41-69)	60 (44-72)
18 - < 40	11 (21)	235 (17.8)
40 - < 50	12 (23)	220 (16.7)
50 - < 60	7 (14)	224 (16.9)
60 - < 70	10 (19)	258 (19.6)
70 - < 80	6 (11.5)	232 (17.6)
≥ 80	6 (11.5)	171 (13.0)
**Female**	31 (59.6)	544 (41.3)
**Obesity**	8 (15.4)	119 (9.0)
**Smoking**	3 (5.8)	83 (6.3)
**Diabetes**	11 (21)	245 (18.6)
**Hypertension**	20 (38.5)	402 (31.0)
**Chronic heart disease**	3 (5.8)	99 (7.5)
**Chronic kidney disease**	3 (5.8)	80 (6.1)
**Immunosuppression**	2 (3.8)	47 (3.6)
**COPD**	14 (27)	103 (8)
**Chronic lung disease**	.	19 (2)
**Homelessness**	2 (3.8)	49 (3.7)
**Uninsured**	42 (81)	1,003 (76)
**Cancer**	7 (13.5)	193 (14.6)
**Number of comorbidities**		
0	14 (27)	565 (43)
1	16 (31)	387 (29)
≥ 2	22 (42)	366 (28)
**RT-PCR**	49 (94)	740 (56)
**Clinical features**	50 (96)	1,298 (99)
**Radiological criteria**	35 (67)	824 (62.5)
ICS use*	44 (85)	101 (8)
**Mild asthma**	48 (92)	.
**Severe asthma**	4 (7.6)	.

IQR = interquartile range; COPD = chronic obstructive pulmonary disease; RT-PCR = real-time polymerase chain reaction; CS = inhaled corticosteroid. * Either continuous use or as relief therapy.

### Severe COVID-19

In a sensitivity analysis considering only patients with positive RT-PCR, the unadjusted estimate for severe COVID-19 among asthmatics remained non-significant (OR 0.84; 95% CI 0.42-1.69).

The proportions of patients in the 1^st^ to 8^th^, 9^th^ to 16^th^ and 17^th^ to 32^nd^ epidemiological weeks were similar between the groups. The need for mechanical ventilation was seen in 36% of the asthmatics and 34% of the non-asthmatics, while death occurred in 23% and 38%, respectively (Table 2).

**Table 2.** Characteristics of hospitalization and outcomes

Variable	Asthmatics (%) n = 52	Non-asthmatics (%) n = 1,318
**Intubation**	19 (36)	444 (34)
**Death**	12 (23)	504 (38)
**Hospitalization (days)**		
< 5	12 (23)	376 (28.5)
5 - < 10	16 (31)	391 (30)
10 - < 15	10 (19)	203 (15.4)
≥ 15	14 (27)	348 (26.4)
**Epidemiological week**		
1-8	18 (35)	265 (20)
9-16	21 (40)	608 (46)
17-32	13 (25)	445 (34)

Asthmatics, compared with non-asthmatics, presented an unadjusted estimate of severe COVID-19 risk of 0.89 (95% CI 0.5-1.56). After multivariable adjustment, the odds ratio (OR) was 0.88 (95% CI 0.5-1.68), i.e. it remained statistically non-significant. Age > 60, male sex, hypertension, diabetes, cancer and homelessness were the covariates associated with a statistically significant increased risk of severe COVID-19. Progression of epidemiological week over the 32-week period was inversely correlated with severe COVID-19 in a statistically significant manner (OR 0.97; 95% CI 0.95-0.98) (Table 3).

**Table 3.** Odds ratio for severe coronavirus disease 2019 (COVID-19)

Variables	Odds ratio	[95% CI]	P value
**Univariate analysis**			
Asthma	0.89	[0.51-1.56]	0.69
**Multivariate analysis**			
Asthma	0.88	[0.5-1.68]	0.7
Age > 60	2.31	[1.8-2.96]	< 0.001
Female	0.63	[0.49-0.8]	< 0.001
Chronic heart disease	1.18	[0.75-1.88]	0.74
Diabetes	1.48	[1.06-2.06]	0.02
Hypertension	1.93	[1.40-2.50]	< 0.001
COPD	1.00	[0.64-1.56]	0.98
Chronic kidney disease	1.31	[0.80-2.16]	0.28
Cancer	2.81	[1.99-3.94]	< 0.001
Smoking	1.71	[0.72-1.90]	0.51
Obesity	1.55	[1.03-2.30]	0.03
Homelessness	2.24	[1.21-4.15]	< 0.001
Chronic lung disease	3.04	[0.69-13.33]	0.14
Epidemiological week	0.97	[0.95-0.98]	< 0.001
ICS*	0.88	[0.46-1.68]	0.71

CI = confidence interval; COPD = chronic obstructive pulmonary disease; ICS = inhaled corticosteroids. *Regular use.

### Overall mortality

Asthmatics’ unadjusted HR for death, in comparison with non-asthmatics, was 0.64 (95% CI 0.7-1.1). After multivariate adjustment, asthmatics presented HR of 0.69 (95% CI 0.39-1.23), in comparison with non-asthmatics. Sensitivity analysis showed HR of 0.68 (95% CI 0.37-1.26) (Figure 1).

Kaplan-Meier estimated survival over hospitalization of up to 30 days showed no difference between the groups (log-rank P = 0.09). The probability of survival among asthmatics, versus non-asthmatics, was respectively 0.88 versus 0.83 at 5 days, 0.77 versus 0.67 at 10 days, 0.65 versus 0.58 at 15 days, 0.65 versus 0.5 at 20 days and 0.65 versus 0.42 at 30 days (Figure 2).

The length of hospitalization was relatively similar in the two groups. 23% of the asthmatics and 28.5% of the non-asthmatics were hospitalized for less than 5 days; 31% of the asthmatics versus 30% of the non-asthmatics were hospitalized for ≥ 5 to < 10 days; and 19% versus 15.4% for ≥ 10 to < 15 days, respectively (Table 2).

## DISCUSSION

In this study, we retrospectively evaluated all patients hospitalized in the first 32 weeks of COVID-19 cases in one of the most important tertiary-level medical centers and philanthropic emergency departments, in São Paulo and in Brazil. The prevalence of asthma was similar to what had been reported previously among COVID-19 patients and to the prevalence in the general population.^10,11^ No statistically significant association was found between asthma and severe COVID-19, and differences in survival were not statistically significant between the groups.

Overall, a pronounced prevalence of comorbidities was observed. This was to be expected, considering that some of the individuals admitted to the emergency department were patients followed up at our institution’s subspecialty outpatient clinics. Hypertension, diabetes and cancer were associated with increased risk of severe COVID-19 and death, as also found in other very recent reports.^11^ In the asthma group, proportionally more individuals with at least two comorbidities and with at least three comorbidities were seen. In contrast, although our institution is a referral hospital for treating severe asthma, patients with this condition were underrepresented. Most of them presented mild asthma and were either in step 1 or step 2 of treatment. This observation was consistent with recent studies, in which patients with severe asthma did not seem to present increased susceptibility to severe forms of SARS-CoV-2 infection.^12^

In addition, we found a positive correlation between severe disease and homelessness, which is a condition of extreme social vulnerability. The incidence of COVID-19 incidence has been correlated with high social vulnerability,^13^ but the extent to which this can affect the course of this disease remains unknown. We observed that women predominated in the asthma group, which is consistent with the epidemiology of asthma, and this contrasted with the predominance of males in the non-asthma group. This pattern has been seen in most COVID-19 cohorts.^11^

Age is a special confounder regarding asthma and worse outcomes from COVID-19. Lung function is overall reduced in elderly asthmatics^14^ and the features of type 2 inflammation, which is supposedly associated with a protective effect against severe COVID-19, may be notably reduced.^15^ Although we did not perform multiple age-stratification, given the relatively small number of asthmatics, we can assume that the impact of aging might give rise to a less protective relationship for asthma against SARS-CoV-2 infection.

Asthma did not have any impact on the time that elapsed until death or the duration of hospitalization, as reported from other observational studies.^11^ Mortality among both asthmatics and non-asthmatics was reduced, given the course of the pandemic in the year 2020, as suggested by other reports.^16^ This was also seen in the decreased odds for severe COVID-19, given the course of the epidemiological weeks.

It remains to be elucidated whether asthma has a protective effect against severe COVID-19. Even through the most recent meta-analyses, this could not be determined.^17,18^ Nonetheless, despite not finding any significant association between asthma and reduced risk of severe COVID-19, we believe that the biological mechanisms associated with asthma have a small but protective role. This could occur either directly, through the phenotypic T-helper cell type 2 (Th2) immune response, or indirectly, through ICS use.

It is possible that different asthma phenotypes might account for different risk relationships with severe COVID-19. A large analysis on polygenic risk scores for asthma phenotypes found that there was a significant risk association for severe COVID-19, driven by non-allergic asthma, which remained significant after stratifying according to presence of COPD.^19^ Accordingly, increased transcript levels of FURIN, a host cell enzyme that enables activation of spike proteins, were found in neutrophilic asthma.^20^ In the present study, we were not able to determine asthma phenotypes, but we can hypothesize that there was high prevalence of non-allergic asthma, given the high proportion of COPD-asthma overlap. This condition is considered to be a non-type 2 inflammatory response and is driven by different cytokines such as interleukin (IL)-8 and IL-17, which are not related to atopy.^20^ In addition, it is known that patients with clinical features of both asthma and COPD present increased overall mortality, in comparison with asthma or COPD alone. A large systematic review and meta-analysis revealed a high-risk association between COPD and severe COVID-19.^17^ Thus, in our sample, the noticeable prevalence of COPD among asthmatics might have biased the association of asthma with severe COVID-19 towards the null hypothesis.

In allergic asthma, downregulation of ACE2 has been associated with a type 2 immune profile,^21,22^ through which increased levels of IL-13 might reduce the expression of disintegrin and metalloprotease 17 (ADAM-17). This protein cleaves the ACE2 protein and facilitates endocytosis of the ACE2–SARS-CoV-2 complex.

Moreover, ICS use has been studied as another underlying mechanism associating asthmatics with reduced ACE2 expression, through suppression of type 1 interferon response^23^ and decreased transcription of genes that are important for virus co-hosting. In a placebo-controlled randomized trial, it was observed that genes that were co-expressed with ACE2, particularly ADAM17 and FURIN, were underexpressed after ICS use.^24^

Our study has many limitations. First, the radiological criteria for SARS-CoV-2 infection were obtained from the medical records, which were elaborated by internists and, as needed, consulting radiologists. Furthermore, patients who were transferred to other hospitals (6% of the initially eligible sample) were excluded due to missing final outcome data. This could have biased the sample towards inclusion of more severe cases. Third, not all of the individuals included had RT-PCR results, especially at the beginning of the pandemic. However, in sensitivity analyses that included only those with RT-PCR positivity for SARS-CoV-2, the results were not significantly altered, which indicates that our findings had good internal and external validity. Overall, the groups were not significantly heterogeneous, except mainly for sex and age. However, the small size of the asthmatics group may have accounted for the non-significant confidence intervals of the association measurements.

One of the strengths of this analysis was that it comprised an evaluation on a large and representative sample of COVID-19 hospitalized patients in São Paulo, the most populous city in Latin America. Furthermore, our main data source came from a tertiary referral hospital that has the largest philanthropic emergency department in Brazil. Thus, our study helps to provide information about severe COVID-19 in a context of a highly comorbid and socially vulnerable population.

## CONCLUSIONS

In summary, asthma was not associated with either decreased odds for severe COVID-19 or increased survival. Future larger studies evaluating ICS use, as well as asthma phenotypes, might be able to point out a possible small, but protective effect of asthma against severe COVID-19. It is of the utmost importance to determine which subgroups and phenotypes are at increased risk of severe COVID-19, in order to guide public healthcare policies. In addition, future investigations of COVID-19 outcomes according to subsets of asthma might provide new insights on SARS-CoV-2 immunopathology.
